# Validation of Deep Learning-Based Artifact Correction on Synthetic FLAIR Images in a Different Scanning Environment

**DOI:** 10.3390/jcm9020364

**Published:** 2020-01-29

**Authors:** Kyeong Hwa Ryu, Hye Jin Baek, Sung-Min Gho, Kanghyun Ryu, Dong-Hyun Kim, Sung Eun Park, Ji Young Ha, Soo Buem Cho, Joon Sung Lee

**Affiliations:** 1 Department of Radiology, Gyeongsang National University School of Medicine and Gyeongsang National University Changwon Hospital, Changwon 51472, Korea; ryukh0329@gmail.com (K.H.R.); uneyes@hanmail.net (S.E.P.); wonpiece@gmail.com (J.Y.H.); 2 Department of Radiology, Institute of Health Sciences, Gyeongsang National University School of Medicine, Jinju 52727, Korea; 3 MR Collaboration & Development, GE Healthcare Korea, Seoul 06060, Korea; 4 Department of Electrical and Electronic Engineering, Yonsei University, Seoul 03722, Korea; nelson666@naver.com (K.R.);; 5 Department of Radiology, Ewha Womans University, College of Medicine, Seoul 07804, Korea; kingnose80@gmail.com; 6 Regional Research, GE Healthcare Korea, Seoul 06060, Korea; Joonsung.Lee@ge.com

**Keywords:** neural networks (computer), deep learning, image enhancement, magnetic resonance imaging, image interpretation, computer-assisted

## Abstract

We investigated the capability of a trained deep learning (DL) model with a convolutional neural network (CNN) in a different scanning environment in terms of ameliorating the quality of synthetic fluid-attenuated inversion recovery (FLAIR) images. The acquired data of 319 patients obtained from the retrospective review were used as test sets for the already trained DL model to correct the synthetic FLAIR images. Quantitative analyses were performed for native synthetic FLAIR and DL-FLAIR images against conventional FLAIR images. Two neuroradiologists assessed the quality and artifact degree of the native synthetic FLAIR and DL-FLAIR images. The quantitative parameters showed significant improvement on DL-FLAIR in all individual tissue segments and total intracranial tissues than on the native synthetic FLAIR (*p* < 0.0001). DL-FLAIR images showed improved image quality with fewer artifacts than the native synthetic FLAIR images (*p* < 0.0001). There was no significant difference in the preservation of the periventricular white matter hyperintensities and lesion conspicuity between the two FLAIR image sets (*p* = 0.217). The quality of synthetic FLAIR images was improved through artifact correction using the trained DL model on a different scan environment. DL-based correction can be a promising solution for ameliorating the quality of synthetic FLAIR images to broaden the clinical use of synthetic magnetic resonance imaging (MRI).

## 1. Introduction

Synthetic magnetic resonance imaging (MRI) is based on a quantitative approach using absolute physical properties such as the longitudinal T1-relaxation time, transverse T2-relaxation time, and proton density [1,2,3,4,5]. It can generate multiple contrast-weighted images in a single scan with modifiable acquisition parameters such as repetition time (TR), echo time (TE), and inversion time (TI) derived from mathematical inferences rather than being predetermined [1,2,3,4,5]. In previous studies, the clinical utility of synthetic MRI was investigated by assessing its image quality and diagnostic performance for detecting a range of brain abnormalities [4,5,6,7,8]. However, synthetic fluid-attenuated inversion recovery (FLAIR) artifacts are major drawbacks limiting the effectiveness of synthetic MRI for clinical use, even though synthetic MRI has a comparable diagnostic performance with that of conventional MRI and can reduce the scan time in the clinical setting [4,5,6,7,8]. It is well-known that synthetic FLAIR artifacts appear as thin, granulated, and marginal hyperintensity along the brain surface [4,5,6,8] or parenchymal swelling in the brain-cerebrospinal fluid (CSF) interface [9], resulting in a decrease in the overall image quality. Therefore, further efforts to improve the image quality of the synthetic FLAIR images are essential to expand the clinical use of synthetic MRI in daily clinical practice.

Two recent studies using deep learning (DL) have introduced the improvement of the synthetic FLAIR image quality [10,11]. Although those studies employed different methodological approaches—convolutional neural network with perceptual loss function (CNN) vs. pixel-wise neural network with conditional generative adversarial network (GAN) loss function—both of them showed remarkable potential to solve this issue [10,11]. However, the studies used DL-based correction for a synthetic FLAIR image employed a limited number of study participants at each institution; therefore, these new approaches should be validated in a different scan environment to establish their clinical utility. Thus, we aimed to investigate the capability of the already-trained DL model with CNN [10] in a different scanning environment from the perspective of ameliorating the image quality of synthetic FLAIR images.

## 2. Patients and Methods

### 2.1. Study Population

A review of our institutional database revealed 321 consecutive patients who underwent routine brain MRI with synthetic acquisition between July and December 2018. Among them, two patients who had not undergone 2D or 3D conventional FLAIR images were excluded. We ultimately enrolled 319 of the 321 patients for this study, comprising 176 men and 143 women with a mean age of 58.7 ± 12.7 years (range, 21–83 years). Of these, 19 patients had 2D FLAIR images, whereas 300 patients had 3D FLAIR images. 

MRI examinations were performed for headache (73/319, 22.9%), dizziness or vertigo (58/319, 18.2%), infarction follow-up (43/319, 13.5%), brain metastasis work-up (37/319, 11.6%), weakness of the extremities (27/319, 8.5%), syncope (25/319, 7.8%), brain tumor follow-up (21/319, 6.6%), sensory change (19/319, 6%), and altered mental status (16/319, 5%).

In the present study, the retrospective data collection and analyses were performed in accordance with the local institutional review board guidelines after obtaining its approval. The institutional review board determined that patient approval and informed consent were not required for retrospectively reviewing images and electronic medical records.

### 2.2. Image Acquisition

MRI was performed using a 3T system (Signa™ Architect; GE Healthcare, Milwaukee, WI, USA) with a 48-channel head coil. All the patients underwent synthetic MRI and conventional 2D or 3D FLAIR imaging. Synthetic MRI was acquired using the multiple-dynamic multiple-echo sequence [3]. The acquisition parameters of the synthetic MRI were as follows: TE = 16.8 and 92.4 ms; delay times = 210, 610, 1810, and 3810 ms; TR = 4000 ms; FOV = 220 × 194 mm; matrix size = 320 × 288; ETL = 16; bandwidth = ±35.71 kHz; slice thickness = 5 mm; resulting in a total acquisition time of 4 min 32 s.

Four synthetic images were retrieved from the SyMRI software (Version 8.0; Synthetic MR, Linköping, Sweden): FLAIR, T1-weighted image, T2-weighted image, and proton density image. The parameters for the image synthesis were as follows: TR/TE/TI = 10,000/118/2566 ms for FLAIR, TR/TE = 500/10 ms for the T1-weighted image, TR/TE = 5000/70 ms for the T2-weighted image, and TR/TE = 3500/30 ms for the proton density image.

The acquisition parameters for the conventional FLAIR imaging were as follows: TR/TE/TI = 10,000/118.5/2566.65 ms, ETL = 27, and acquisition time = 2 min 7 s for the 2D FLAIR image, other parameters such as FOV or resolution were equivalent to the synthetic images; and TR/TE/TI = 6800/105/1912 ms, ETL = 180, FOV = 250 × 250 mm; matrix size = 220 × 220; ETL = 16; bandwidth = ±62.5 kHz; slice thickness = 1.2 mm; and acquisition time = 3 min 22 s for the 3D FLAIR image.

### 2.3. DL Framework

To apply the DL-based artifact correction in the present study, a pretrained CNN from an original work by Ryu et al. [10] was used. The network architecture was based on the residual nets (RESNET) architecture [12] with several modifications. This network used two combined loss functions, namely the mean absolute error and perceptual loss [13]. While this DL-based method has shown promising results in correcting artifacts in synthetic FLAIR, the previous validation study of the method relied on a dataset from a single scanner and only a small number of test data for 20 subjects [10]. Moreover, the network of the previous study was entirely trained on images obtained from a single scanner (GE Discovery 750W GE Healthcare, Milwaukee, USA) in a single institution [10].

For additional validation in this study, the network was tested in a different environment from our institution. The data for this study was obtained from a different scanner (Signa™ Architect; GE Healthcare, Milwaukee, WI, USA) and using a different number of receiver coils (48-channel head coil). Note that no additional training was performed in the current study. The imaging protocols of training and test sets were similar in terms of TE/TR, slice thickness, decay times, and bandwidth. In addition, the image voxel of the test set in the current study was 40% smaller than that of the training set.

For the subjects in the current study, the forward-pass of the network was used to produce the DL corrected images. This process was repeated slice by slice. It took approximately 1.1 s per subject for 25 slices to obtain the output images. After the completion of the process for a subject, the native synthetic FLAIR (input), DL-FLAIR (output), and conventional 2D or 3D FLAIR images were stored separately for evaluation.

The testing was performed using a single GPU (NVIDIA TITAN XP) with the Keras framework [14] and a TensorFlow [15] backend, CUDA 10.0, and CUDNN 7.1 on a Linux server.

### 2.4. Image Analyses of Native and Corrected Synthetic FLAIR Images

#### 2.4.1. Quantitative Analyses

Of the 319 patients, the quantitative analyses were performed for 19 patients who had available conventional 2D FLAIR images for a direct comparison of the region-wise evaluation. For the quantitative evaluation, normalized root mean squared error (NRMSE), peak signal-to-noise ratio (PSNR), and structural similarity index (SSIM) were used. The NRMSE measures the normalized voxel-wise intensity differences (errors) while the SSIM measures the nonlocal structural similarity. PSNR was calculated by the following equation:

$$PSNR=20\;\log_{10}\frac{25,500}{\sqrt{\mathrm{MSE}}}$$

where 25,500 is the maximum range of the FLAIR signal intensity.

**Figure 1:**
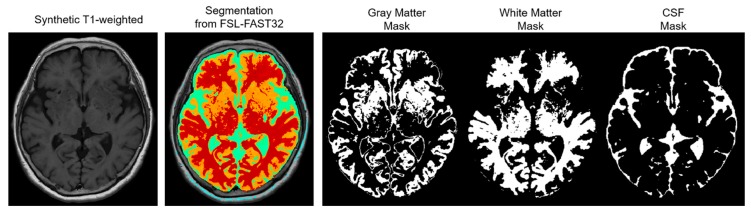
Illustration of the segmented regions during the region-wise evaluation. The region-wise evaluation is based on three automatically segmented regions: gray matter, white matter, and cerebrospinal fluid (CSF). The segmentations for the regions are retrieved via segmentation with FSL-FAST32 using the synthetic T1-weighted images.

The NRMSE and PSNR were compared based on three automatically segmented regions as follows: gray matter (GM), white matter (WM), and CSF. This region-wise evaluation was conducted to indicate which region was most improved, and the segmentations for the regions were retrieved via segmentation with FSL-FAST32 using the synthetic T1-weighted images (Figure 1).

#### 2.4.2. Qualitative Analyses

All the datasets were anonymized, and the reader reviewed all images using the picture archiving and communication system. Two attending neuroradiologists, having nine and four years of experience, performed independent analyses of the native synthetic FLAIR and DL-FLAIR images of all 319 patients according to assessment criteria of each item listed in Table 1. The synthetic FLAIR and DL-FLAIR images were assessed in random order after mixing the two FLAIR image sets to minimize bias. The image analyses were performed twice by each reader with a memory wash-out period of two weeks. In each session, the order of review of the studies was random.

### 2.5. Statistical Analysis

The data were tested for normal distribution using the Kolmogorov–Smirnov test. Paired t-tests were performed on the quantitative assessment results. For qualitative results, the scores of each image set from the two readers were averaged, and the Wilcoxon signed-rank test was conducted to compare the scores of synthetic FLAIR and DL-FLAIR images. Interobserver agreement between two readers was calculated using weighted kappa statistics. According to the recommendation by Landis and Koch [16], the weighted kappa value was interpreted as follows: 0, no agreement; 0.01–0.20, slight agreement; 0.21–0.40, fair agreement; 0.41–0.60, moderate agreement; 0.61–0.80, substantial agreement; and 0.81–1.00, almost perfect agreement. All the statistical analyses were conducted using SPSS, version 24.0 (IBM Corp., Armonk, NY, USA), and the statistical significance was set at *p* < 0.05 (two-sided).

## 3. Results

Of 319 patients, 203 (63.6%) had abnormal MRI findings and 116 (36.4%) had normal findings. The following diagnoses were made: ischemic/hemorrhagic stroke or small vessel disease (154/203, 75.9%), intracranial neoplasm (18/203, 8.9%), vascular abnormality (11/203, 5.4%), infectious or demyelinating disease (7/203, 3.4%), metabolic or degenerative disorder (4/203, 2%), and miscellaneous, including trauma or indeterminate condition (9/203, 4.4%).

**Figure 2:**
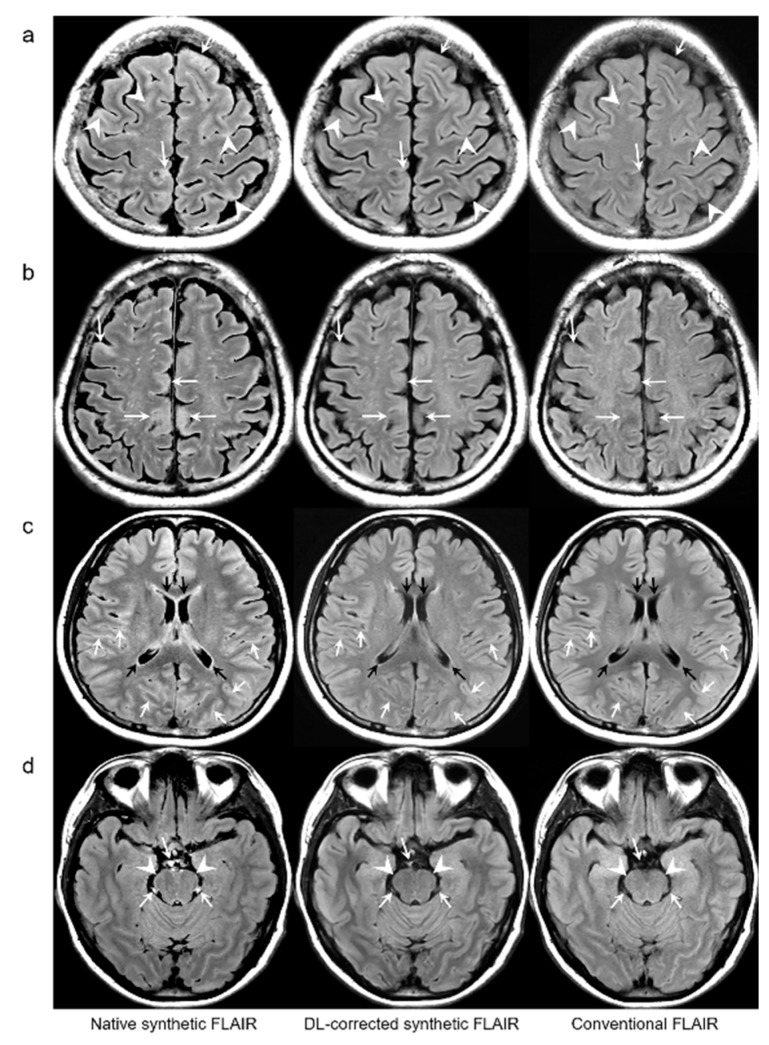
Representative results of synthetic fluid-attenuated inversion recovery (FLAIR) artifact reduction. Pairs of native synthetic FLAIR, deep learning (DL)-FLAIR, and conventional FLAIR images are shown (a–d). (**a**) Surface hyperintensity artifacts (arrowheads) and cortical swelling artifacts (arrows) on synthetic FLAIR are almost removed on DL-FLAIR image. (**b**) Cortical swelling artifacts (arrows) on native synthetic FLAIR are successfully removed on DL-FLAIR image. (**c**) Periventricular hyperintense artifacts along the margin of bilateral lateral ventricles (black arrows) and surface hyperintensity artifacts (white arrows) on the native synthetic FLAIR are successfully eliminated on DL-FLAIR image. (**d**) Flow artifacts in the prepontine cistern (white arrows) and surface hyperintensity artifacts along the pons (white arrowheads) on the native synthetic FLAIR are improved on DL-FLAIR image.

**Figure 3:**
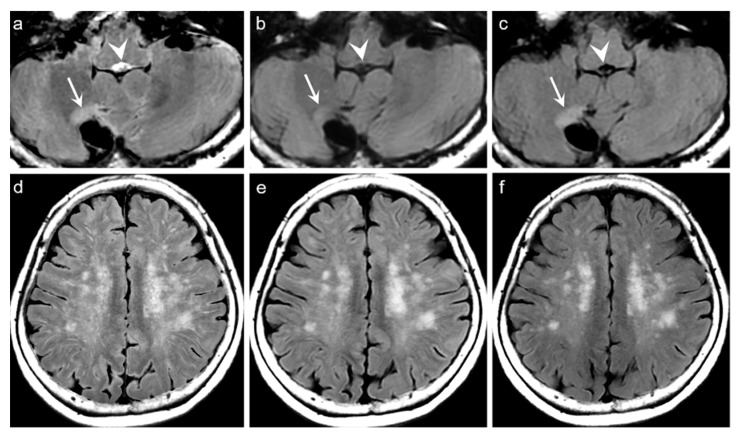
Representative case of lesion conspicuity on DL-FLAIR images. Focal marginal gliosis showing hyperintensity (arrows) is seen at the anterior aspect of the surgical cavity in the right cerebellum (a–c). The lesion is well delineated on the native synthetic FLAIR (**a**), DL-FLAIR (**b**), and conventional FLAIR images (**c**). However, the hyperintense lesion on the native synthetic FLAIR (arrow on a) is incompletely preserved on DL-FLAIR image (arrow on b), showing a decrease in the degree of its hyperintensity. Flow artifacts in the fourth ventricle on the native synthetic FLAIR (arrowhead on a) are successfully removed on DL-FLAIR image (arrowhead on b). Multiple FLAIR hyperintense lesions in both centrum semiovale, suggesting grade II small vessel disease on the native synthetic FLAIR (**d**) are well preserved on DL-FLAIR image (**e**). (**f**) Conventional FLAIR image is shown for comparison.

**Figure 4:**
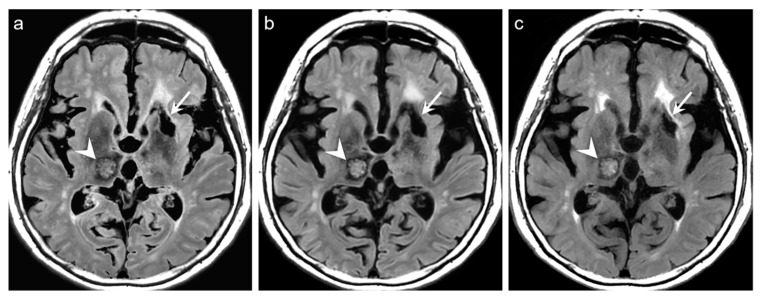
Representative case of lesion conspicuity on DL-FLAIR image. Intracranial hemorrhage (ICH) (arrowheads) is well visualized in the right thalamus on the native synthetic FLAIR (**a**), DL-FLAIR (**b**), and conventional FLAIR images (**c**). In particular, the thalamic ICH is more conspicuously delineated on DL-FLAIR (**b**) than on the native synthetic FLAIR image (**a**). However, the conspicuity of the true hyperintensity around the encephalomalacia in the left basal ganglia (arrows on a, c) is slightly decreased on DL-FLAIR image (arrow on b).

**Figure 5:**
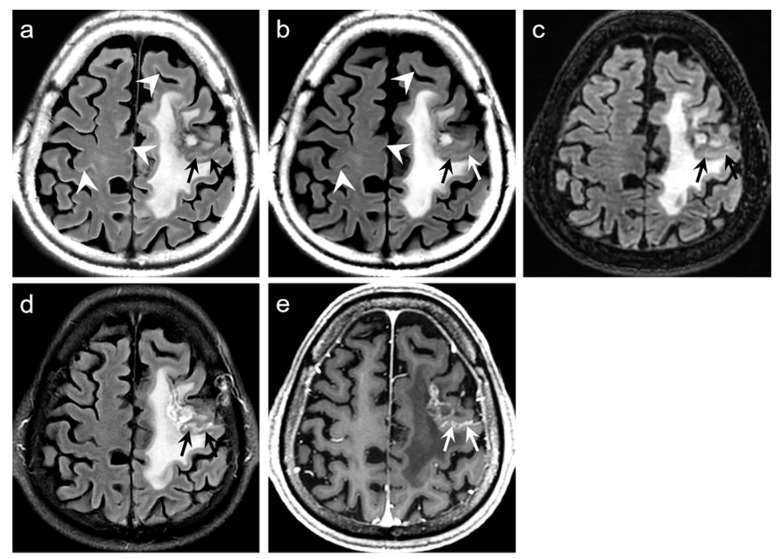
Representative case of preservation of true sulcal lesion in patient with brain and leptomeningeal metastases from lung cancer. Leptomeningeal metastases showing sulcal hyperintensity (arrows) on the native synthetic FLAIR (**a**) are preserved on DL-FLAIR (**b**), whereas surface hyperintense artifacts on the native synthetic FLAIR (arrowheads on a) are removed on DL-FLAIR image (**b**). The leptomeningeal metastases are conspicuously delineated on 3D-FLAIR (**c**), and the corresponding lesions show prominent enhancement on Gd-enhanced FLAIR (**d**) and Gd-enhanced 3D T1-weighted image (**e**).

Representative examples are depicted in Figure 2, Figure 3, Figure 4 and Figure 5. Table 2 summarizes the results of the quantitative assessment of NRMSE, PSNR, and SSIM for native synthetic and DL-FLAIR images calculated against the conventional 2D FLAIR images of 19 patients. Theoretically, images with a lower NRMSE, higher PSNR, and higher SSIM indicate better image quality. In this study, all values of NRMSE, PSNR, and SSIM were improved by the DL-based correction of the synthetic FLAIR images. The NRMSE was significantly lower for DL-FLAIR than for the native synthetic FLAIR images in GM, WM, CSF, and total intracranial tissues (all *p* < 0.0001). The NRMSE of the synthetic and DL-FLAIR images was the highest in CSF, with GM showing higher values than WM (all *p* < 0.0001). However, the percent change in NRMSE was the highest in GM, followed by CSF and WM. The PSNR was significantly higher for DL-FLAIR than for native synthetic FLAIR images in GM, WM, CSF, and total intracranial tissues (all *p* < 0.0001). The PSNR was the lowest in CSF, with GM showing lower values than WM in both DL-FLAIR and native synthetic FLAIR images (all *p* < 0.0001). In contrast, the percent change in PSNR was the highest in CSF, followed by GM and WM. In addition, the SSIM is improved from 0.907 to 0.938 (*p* < 0.0001). For the region-wise NRMSE and PSNR values, the improvement was more distinctive in GM and CSF than in WM.

For the qualitative analyses of 319 patients, the mean scores of both DL-FLAIR and native synthetic FLAIR images showed acceptable image quality for diagnostic use. The qualitative assessment scores given by the two readers and the corresponding interobserver reliability are shown in Table 3. The average mean scores of DL-FLAIR image quality were significantly higher than those of the image quality of the native synthetic FLAIR (4.73 ± 0.46 vs. 3.12 ± 0.69; *p* < 0.0001). The average mean scores of the degree of preserving the preexisting periventricular WM hyperintensities or lesion conspicuity were not statistically significant for DL-FLAIR and native synthetic FLAIR: 4.69 ± 0.68 vs. 4.70 ± 0.61 (*p* = 0.217). Among the 319 patients, there was no case of generation of artificial pseudolesions during DL processing. However, it was possible to identify incomplete preservation of the preexisting true hyperintensities on DL-FLAIR images in 11 patients (3.4%) among 319 patients owing to the unexpected partial removal of the true hyperintensities (Figure 3b and Figure 4b). The mean scores of the typical synthetic FLAIR artifacts including surface hyperintensities, granularities, or cortical swelling were identified for DL-FLAIR and native synthetic FLAIR images as follows: 1.32 ± 0.51 vs. 3.35 ± 0.68 (*p* < 0.0001) (Figure 2). In addition, other artifacts that substantially degraded the image quality, such as flow artifacts, were also improved in DL-FLAIR rather than in the native synthetic FLAIR: 1.27 ± 0.46 vs. 2.43 ± 0.72 (*p* < 0.0001) (Figure 2d).

## 4. Discussion

The findings of our study indicate that artifact correction using an already-trained DL algorithm could improve the image quality of synthetic FLAIR images by successfully removing native artifacts from an external data set in a different scanning environment, and it could also provide significantly better values of quantitative parameters. In addition, to the best of our knowledge, this is the first study to employ such a large sample size for the external validation of the trained DL model and provide three quantitative parameters for evaluating the image quality of DL-FLAIR and native synthetic FLAIR images.

In previous studies, synthetic FLAIR artifacts did not have a significant effect on the diagnosis because the artifacts could easily be differentiated among the pathologic conditions [5,8]. However, synthetic FLAIR artifacts are an issue for routine clinical use because they can mimic a pathology in the CSF-filled spaces or CSF–brain interface; to identify them, radiologists should undergo an adaptation period to gain familiarity with this issue. Thus far, the exact cause of such artifacts remains unclear; however, it may be related to the partial volume and flow effects from previous studies [4,5,10]. Fortunately, synthetic FLAIR artifacts have characterized patterns, thin, granulated, and marginal hyperintensity along the brain surface and CSF spaces, and they tend to appear in high convexities and posterior compartments, such as temporo-occipital regions and the brainstem. Therefore, DL-based artifact correction can improve the image quality of the synthetic FLAIR images.

Recently, DL methods have been applied increasingly in the field of radiology, and they have demonstrated enormous potential in several MRI processing areas [17], including artifact correction for specific pulse sequences [18,19]. Thus, recent studies have developed DL algorithms using variants of CNN to remove synthetic FLAIR artifacts and have thus demonstrated the feasibility of this method. However, two studies presented limitations because they were conducted using the same 3T MR scanner provided by a single vendor, although the institutions were different [10,11]. Therefore, our results are promising for generalizing the application of the DL method for improving synthetic FLAIR image quality because overfitted DL models only work for internal datasets and exhibit poor performance for external datasets [20].

The results of the current study also revealed that the DL algorithm using CNN improved the image quality of the synthetic FLAIR images by correcting the typical artifacts in both quantitative and qualitative analyses, and it is consistent with the results of two recent studies [10,11]. In the current study, both NRMSE and PSNR values in the DL-FLAIR image were more distinctive in GM and CSF regions than in WM in the region-wise analyses, which is consistent with the quantitative analysis of the recent study [10]. This may indicate that our DL-based artifact correction mainly acted on the brain surface and CSF spaces, which are the most common locations of synthetic FLAIR artifacts. Therefore, these results show the potential for the application of synthetic MRI in clinical use by enabling accurate detection of true intracranial pathologies at the brain–CSF interface on the synthetic FLAIR images. In addition, the improvement shown in the quantitative analysis was the lowest in WM, with no significant difference noticed in the degree of preserving the preexisting periventricular WM hyperintensities or lesion conspicuity on the visual assessment for DL-FLAIR images. The reason for this is unclear; therefore, additional studies are required to investigate this issue by comparing native synthetic FLAIR, DL-FLAIR, and conventional FLAIR images for expanding the diagnostic use of synthetic MRI in daily clinical practice.

In terms of image artifacts, the typical synthetic FLAIR artifacts were significantly improved in DL-FLAIR images (Figure 2), which is consistent with the original work [10]. The CNN used in the current study is well-known for being highly effective in sensing and learning spatial patterns or features [20,21]. Fortunately, synthetic FLAIR artifacts have characterized patterns showing granulated and marginal hyperintensity along the brain-CSF interface [4,5,8,10]. Therefore, our DL method enables the efficient detection and removal of artifacts according to their spatial patterns. However, further comparative studies using different DL methods should be conducted to investigate the effectiveness and differences to reduce synthetic FLAIR artifacts.

In the present study, we could identify the incomplete preservation of pre-existing true hyperintensities on DL-FLAIR images in 11 patients, owing to the unexpected partial removal of the true hyperintensities. In all these cases, the hyperintense lesions were located in the vicinity of cystic encephalomalacias, and the lesions were considered to be reactive gliosis. The reason for this finding is unclear; however, it may be related to the processing of DL-based artifact correction to distinguish artifacts from true hyperintensities, especially when the true hyperintensities were seen near fluid-containing lesions, making the fluid-lesion interface likely to be similar to the CSF-brain parenchyma interface. We believe that the issue can be solved if the DL algorithm is improved through a further training process using various pathologic cases that can differentiate a normal CSF-tissue interface from the lesion-fluid interface.

Although the results are promising, this study has certain limitations. First, we did not directly compare the qualities of DL-FLAIR and conventional FLAIR images because this study included two types of conventional FLAIR images: 2D and 3D. This was unavoidable because this study was retrospectively designed. We believe that further studies should be required to directly compare the image quality of DL-FLAIR and conventional FLAIR images for attesting the clinical use of DL-FLAIR images and validating our results. Second, the quantitative analyses were performed only for 19 patients owing to the mentioned heterogeneity of the conventional FLAIR images. Third, we obtained all data by using a different MR scanner with different scan parameters in a single institution; however, previous studies used scanners from the same vendor [10,11]. Therefore, we expect that future studies with different scanners from other vendors in multiple institutions will be conducted to validate and generalize our results. Finally, we did not perform a meticulous evaluation of the intracranial pathologies during the analyses because we focused on the improvement of the synthetic FLAIR image quality by DL-based correction and the heterogeneous brain MRI protocols related to the patients’ medical condition.

## 5. Conclusions

In conclusion, the artifact correction with the already-trained DL algorithm led to successful improvements in the image quality of the synthetic FLAIR images upon usage of an external dataset on a different MR scanner in different scan environments. This was verified both qualitatively and quantitatively, and the obtained images were compared with the conventional FLAIR images. Therefore, we believe that the DL-based approach can provide a promising solution for improving the image quality of synthetic FLAIR images to broaden the clinical use of synthetic MRI in daily clinical practice.

## Figures and Tables

**Table 1:** Assessment criteria for qualitative analysis using the Likert scale.

Criteria Assessed	Assessment Scale
Image quality	(1) Non-diagnostic
	(2) Bad (not acceptable for diagnostic use)
	(3) Acceptable (acceptable for diagnostic use but with minor issues)
	(4) Good (acceptable for diagnostic use)
	(5) Excellent (acceptable for diagnostic use)
Preservation of the periventricular white matter hyperintensities or lesion conspicuity	(1) Extremely poor
	(2) Poor
	(3) Acceptable
	(4) Good
	(5) Excellent
Degree of typical synthetic FLAIR artifacts * and other artifacts ^+^	(1) None or negligible
	(2) Mild (less than 30% of the axial images)
	(3) Moderate (between 30%–50% of the axial images)
	(4) Severe (above 50% of the axial images)

FLAIR, fluid-attenuated inversion recovery. * Typical synthetic artifacts are surface hyperintensity, granular artifact, or cortical swelling artifact. ^+^ The degree of other artifacts that substantially degraded the image quality through, for example, flow artifact, were also assessed.

**Table 2:** NRMSE, PSNR, and SSIM of native synthetic FLAIR and DL-FLAIR images against conventional 2D FLAIR images in various regions in 19 patients.

	Native Synthetic FLAIR Images	DL-FLAIR Images
GM		
NRMSE	0.214 ± 0.076 *^+^	0.128 ± 0.016 (−40.187%) *^+^
PSNR	47.654 ± 2.520 *^+^	51.950 ± 2.239 (+9.015%) *^+^
WM		
NRMSE	0.093 ± 0.031 *^+^	0.085 ± 0.007 (−8.602%) *^+^
PSNR	54.895 ± 2.641 *^+^	55.705 ± 2.582 (+1.476%) *^+^
CSF filled spaces		
NRMSE	0.481 ± 0.230 *^+^	0.297 ± 0.089 (−38.254%) *^+^
PSNR	45.386 ± 1.821 *^+^	49.485 ± 2.122 (+9.031%) *^+^
Total intracranial tissues		
NRMSE	0.202 ± 0.055 *	0.134 ± 0.018 (−33.663%) *
PSNR	48.907 ± 2.031 *	52.442 ± 2.120 (+7.228%) *
SSIM for total intracranial tissues	0.907 ± 0.040 *	0.938 ± 0.030 (+ 3.418%) *

Values are means ± standard deviation. Percentage changes in NRMSE, PSNR, and SSIM for DL-FLAIR vs. native synthetic FLAIR are in parentheses. CSF, cerebrospinal fluid; DL, deep learning; FLAIR, fluid-attenuated inversion recovery; GM, gray matter; NRMSE, normalized root mean squared error; PSNR, peak signal-to-noise ratio; SSIM, structural similarity index; WM, white matter. * *p* < 0.0001 for native synthetic FLAIR vs. DL-FLAIR images. ^+^ *p* < 0.0001 for GM vs. WM, GM vs. CSF, and WM vs. CSF.

**Table 3:** Qualitative assessment of each reader on DL-FLAIR and native synthetic FLAIR images in all 319 patients.

	DL-FLAIR				Native Synthetic FLAIR			
	Reader 1 (Mean ± SD)	Reader 2 (Mean ± SD)	Agreement (κ value)	p value ^#^	Reader 1 (Mean ± SD)	Reader 2 (Mean ± SD)	Agreement (κ value)	p value ^#^
Image quality	4.72 ± 0.48	4.74 ± 0.46	0.834	<0.001	3.11 ± 0.68	3.07 ± 0.63	0.817	<0.001
Degree of conspicuity *	4.68 ± 0.63	4.69 ± 0.61	0.961	<0.001	4.71 ± 0.64	4.70 ± 0.67	0.956	<0.001
Synthetic FLAIR artifacts ^+^	1.30 ± 0.46	1.33 ± 0.49	0.897	<0.001	3.38 ± 0.72	3.32 ± 0.71	0.794	<0.001
Other artifacts ^‡^	1.25 ± 0.41	1.28 ± 0.43	0.876	<0.001	2.38 ± 0.77	2.48 ± 0.74	0.823	<0.001

DL, deep learning; FLAIR, fluid-attenuated inversion recovery. * Degree of conspicuity are degrees of preservation of the periventricular white matter hyperintensities or lesion conspicuity. ^+^ Synthetic FLAIR artifacts are surface hyperintensity, granular artifact, or cortical swelling artifacts. ^‡^ Other artifacts are artifacts that substantially degraded the image quality through, for example, flow artifact. ^#^ *p* values are derived from the kappa statistics.
